# Impact of Implementing a Multidisciplinary Enhanced Recovery Program on Hospital Stay for Patients Undergoing Radical Prostatectomy: A Retrospective Cohort Study

**DOI:** 10.7759/cureus.81772

**Published:** 2025-04-05

**Authors:** Roberto González, Nicolás Valls, Nicolás Villablanca, Roberto Vilches

**Affiliations:** 1 Anesthesiology, National Cancer Institute, Santiago, CHL; 2 Anesthesiology and Perioperative Medicine, University of Chile Clinical Hospital, Santiago, CHL; 3 Anesthesiology, National Cancer Institute, Santigo, CHL; 4 Anesthesiology, Clínica Universidad de los Andes, Santiago, CHL; 5 Urology, National Cancer Institute, Santiago, CHL

**Keywords:** enhanced recovery after surgery (eras), length of hospital stay, perioperative care, postoperative complications, radical prostatectomy

## Abstract

Background

Enhanced recovery after surgery (ERAS) protocols are evidence-based, multidisciplinary approaches designed to minimize perioperative stress, accelerate patient recovery, and reduce healthcare resource utilization. Although well-established in colorectal and other major abdominal surgeries, evidence supporting their effectiveness in laparoscopic radical prostatectomy (LRP) remains limited. This retrospective cohort study evaluated whether implementing an enhanced recovery program (ERP), specifically adapted for LRP, would reduce hospital length of stay (LOS) and maintain patient safety.

Methods

A total of 60 patients undergoing radical prostatectomy were retrospectively analyzed and divided into three cohorts: a historical control group (pre-ERP, n=20), an implementation group (ERP compliance <70%, n=20), and a consolidated group (ERP compliance >70%, n=20). Primary outcome measures included hospital LOS, while secondary outcomes encompassed postoperative complications graded by Clavien-Dindo classification, protocol compliance rates, readmissions, and reoperations.

Results

Median hospital LOS significantly decreased following ERP implementation, from five days in the historical group to three days (p<0.001) in the implementation cohort, and further declined to two days (p<0.001) in the consolidated cohort. Higher ERP compliance (>70%) was strongly associated with shorter hospitalization. Importantly, ERP implementation did not increase the incidence of severe postoperative complications (Clavien-Dindo grades III-V), readmissions, or reoperations.

Conclusions

Implementing a structured ERP for laparoscopic radical prostatectomy significantly reduced hospital LOS without negatively impacting patient safety. Higher compliance with ERP elements was strongly correlated with enhanced patient outcomes. These findings highlight the clinical benefits of ERP adoption and underscore the importance of multidisciplinary adherence for optimizing surgical recovery and healthcare efficiency.

## Introduction

Enhanced recovery after surgery (ERAS) protocols emerged in Europe during the 1990s as innovative, multidisciplinary strategies aimed at minimizing perioperative stress, reducing postoperative complications, and expediting patient recovery through evidence-based interventions [1,2]. Initially developed and validated extensively in colorectal surgery [3], ERAS principles have progressively expanded to include various surgical specialties, consistently demonstrating reductions in hospital length of stay (LOS), complication rates, and healthcare costs [4,5].

In urologic oncology, especially in bladder cancer surgery, robust consensus guidelines for ERAS have been established, incorporating multimodal strategies such as preoperative nutritional optimization, minimally invasive surgical techniques, targeted anesthetic protocols, multimodal analgesia, and early postoperative mobilization [6,7]. The implementation of these protocols in urological procedures has consistently yielded improved perioperative outcomes, including reduced morbidity and shortened LOS, underscoring their potential for broader applications [7].

Prostate cancer remains one of the most prevalent malignancies affecting men globally [8]. Over recent decades, surgical management of prostate cancer has shifted significantly toward minimally invasive approaches, notably laparoscopic radical prostatectomy (LRP). Despite this paradigm shift and the proven effectiveness of ERAS in other abdominal and urological procedures, evidence regarding ERAS specifically tailored for LRP is still limited, with the ERAS Society yet to publish formal guidelines for prostatectomy [9,10]. Although LRP generally exhibits lower complication rates and reduced morbidity compared to more extensive procedures such as radical cystectomy or colorectal resection, the potential advantages of ERAS protocols in enhancing patient-centered outcomes, recovery, and overall healthcare efficiency remain substantial.

Given this context, the direct applicability and efficacy of ERAS protocols in radical prostatectomy warrants thorough investigation. Our study aims to evaluate the clinical impact of implementing a multidisciplinary ERP specifically adapted to LRP, comparing outcomes between traditional perioperative care and ERP-driven management. We hypothesize that implementing a structured ERP protocol will result in a significant reduction in hospital LOS without compromising patient safety, measured through complication rates, readmissions, and reoperations.

## Materials and methods

We conducted a retrospective cohort study at the National Cancer Institute to compare the LOS of patients undergoing radical prostatectomies under the ERP with those receiving traditional perioperative care. A secondary objective was to assess the efficacy and safety of the ERP-driven approach. Prior to commencing the study, ethical approval was secured from the Metropolitan East Health Service Ethics Committee (Approval N°: CEC-SSMO 20-09-2022). 

The cohort comprised 40 consecutive patients enrolled in the ERP program from April 2021 to December 2023. This cohort was contrasted with a sample of 20 patients who received treatment before the program’s introduction, spanning from June 2019 to November 2020. For analytical clarity, we subdivided the participants into three distinct groups: the historical cohort (patients treated before ERP implementation), the implementation cohort (patients with ERP compliance below 70%), and the consolidated cohort (patients with a compliance rate exceeding 70% of the ERP).

We adopted the Clavien-Dindo classification, a straightforward and widely accepted system for grading postoperative complications. Originally proposed by Clavien et al. [11] and later refined by Dindo et al. in 2004 [12], this classification provides a clear and systematic approach to assessing surgical complications based on the required therapeutic interventions. It categorizes complications into five distinct grades according to their severity and the level of intervention needed.

Demographic and clinical data were retrospectively extracted from electronic medical records, including patient age, comorbidities, prostate volume, surgical technique, operative duration, blood loss, anesthetic techniques, and protocol adherence rates.

Due to the retrospective study design, no formal sample size calculation was performed. Continuous variables were tested for normality using the Shapiro-Wilk test. Normally distributed continuous variables are presented as mean ± standard deviation (SD), whereas non-normally distributed variables are presented as median and interquartile range (IQR). Categorical variables are summarized using absolute numbers and percentages.

Comparisons between cohorts for categorical variables were conducted using generalized Fisher’s exact test. For continuous variables, we applied the Kruskal-Wallis test for non-parametric distributions and one-way ANOVA for parametric distributions. Post-hoc analyses were performed using the Dunn test with Bonferroni correction or Fisher’s exact tests for pairwise comparisons, as applicable. A two-tailed p-value <0.05 was considered statistically significant.

All statistical analyses and visualizations were conducted using R version 4.2.2 within RStudio (RStudio Team, Boston, MA).

## Results

A total of 60 patients were analyzed, evenly distributed across three cohorts: historical, implementation, and consolidated (n=20 per cohort). The mean patient age was comparable among groups: 62.1 ± 5.2 years (historical), 62.4 ± 7.1 years (implementation), and 63.8 ± 7.2 years (consolidated). The median American Society of Anesthesiologists Physical Status (ASA-PS) classification was uniformly class 2 (IQR: 0) across all cohorts. Hypertension was the most prevalent comorbidity, affecting 60% (n=12), 70% (n=14), and 50% (n=10) of patients in historical, implementation, and consolidated cohorts, respectively. The prevalence of type 2 diabetes mellitus was similar across groups (historical 15%; implementation 10%; consolidated 15%). Median prostate volume did not differ significantly between cohorts [historical: 47 ml (IQR 23); implementation: 48 ml (IQR 28); consolidated: 40 ml (IQR 25)] (Table 1).

**Table 1:** Baseline demographic characteristics of the study cohorts. Data are presented as mean (± standard deviation, SD) for normally distributed continuous variables, median (interquartile range, IQR) for non-normally distributed variables, or number (%) for categorical variables. Comparative analyses across cohorts were conducted employing the generalized Fisher’s exact test for categorical variables. Subsequent post-hoc pairwise comparisons were conducted utilizing the two-group Fisher’s exact test following a significant outcome of the generalized Fisher’s exact test. The Kruskal–Wallis test for non-parametric continuous variables. Significant differences (p<0.05) versus the historical cohort are indicated by superscript “a”. COPD: chronic obstructive pulmonary disease; ASA-PS: American Society of Anesthesiologists Physical Status.

Characteristic	Historical cohort	Implementation cohort	Consolidation cohort	p-value
Number of patients	20	20	20	-
Average age (SD)	62.1 (5.2)	62.4 (7.1)	63.8 (7.2)	0.058
Hypertension nº (%)	12 (60)	14 (70)	10 (50)	0.480
ASA-PS (IQR)	2 (0)	2 (0)	2 (0)	1.000
Diabetes Mellitus nº (%)	3 (15)	2 (10)	3 (15)	0.924
Insulin Resistance nº (%)	3 (15)	2 (10)	1 (5)	0.977
Coronary Heart Disease nº (%)	0	1(5)	0	0.994
Heart Failure nº (%)	0	0	0	-
Chronic Liver Disease nº (%)	0	0	1 (5)	0.994
Obesity nº (%)	6 (30)	6 (30)	4 (20)	0.814
COPD nº (%)	1 (5)	0	0	0.994
Asthma nº (%)	0	1 (5)	1 (5)	0.215
Psychiatric Illness nº (%)	2 (10)	0	2 (10)	0.998
Smoking nº (%)	5 (25)	5 (25)	5 (25)	1.000
Alcohol consumption nº (%)	5 (25)	8 (40)	13 (65)^a^	0.049
Drug use nº (%)	0	0	1 (5)	0.994
Prostate Volume median (IQR)	47 (23)	48 (28)	40 (25)	0.153

Regarding surgical technique, all historical cohort patients underwent open prostatectomy, whereas the implementation and consolidated cohorts exclusively received laparoscopic radical prostatectomy (p<0.001). Surgical duration significantly increased from a median of 150 minutes (IQR: 32) in the historical cohort to 240 minutes (IQR: 96) in the implementation cohort and 205 minutes (IQR: 62) in the consolidated cohort (p=0.047). Mean estimated intraoperative blood loss decreased significantly from 750 ml (±600 ml) in the historical cohort to 300 ml (±200 ml) in the implementation cohort and 200 ml (±162 ml) in the consolidated cohort (p=0.002).

Anesthetic management differed markedly between cohorts. All patients in the historical cohort received general anesthesia combined with epidural analgesia. In contrast, the ERP cohorts predominantly underwent general anesthesia alone (implementation 70%; consolidated 75%), with intrathecal morphine selectively administered in 30% and 25% of the implementation and consolidated cohorts, respectively (Table 2).

**Table 2:** Intraoperative and Anesthetic Variables by Cohort. Data presented include the number (%) for categorical variables, median (IQR) for non-parametric continuous variables, and mean (±SD) for parametric continuous variables. Statistical significance across cohorts was determined by generalized Fisher’s exact test, Kruskal–Wallis test, or one-way ANOVA, respectively. Significant differences (p<0.05) compared with the historical cohort are indicated by superscript “a”.

Intraoperative variables	Historical cohort	Implementation cohort	Consolidation cohort	p-value	
Number of patients	20	20	20	-	
Laparoscopic surgery nº (%)	0	20 (100)^a^	20 (100)^a^	0.001	
Open surgery nº (%)	20 (100)	0^a^	0^a^	0.001	
Median surgery duration minutes (IQR)	150 (32)	240 (96)	205 (62)	0.047	
Average fentanyl dose (SD)	310 (135)	405 (72)	398 (81)	0.073	
Median morphine dose (IQR)	0.2 (1.1)	3.9 (3.7)	2.4 (2.7)	0.112	
Median bleeding (IQR)	650 (600)	300 (225)^a^	250 (137)^a^	0.002	
General anesthesia nº (%)	0	14 (70)^a^	15 (75)^a^	0.001	
General & Regional anesthesia nº (%)	20 (100)	6 (30)^a^	5 (25)^a^	0.008	
Use of intrathecal morphine nº (%)	0	6 (30)^a^	5 (25)^a^	0.028	

Compliance with ERP interventions significantly improved throughout the implementation phases. Adherence to preoperative counseling rose from 0% in the historical cohort to 40% in the implementation cohort, achieving 100% compliance in the consolidated cohort (p<0.001). Similar trends were observed in compliance with a two-hour clear fluid fasting interval and preoperative carbohydrate loading, increasing from 0% historically to 10% during implementation and 100% in the consolidated cohort (p<0.001). Drain avoidance increased from 0% historically to 60% and 85% in implementation and consolidated cohorts, respectively (p<0.001). Early postoperative feeding compliance improved from 50% (historical) and 55% (implementation) to 90% (consolidated; p=0.016). Additionally, early mobilization within eight hours postoperatively increased markedly from 0% historically to 35% during implementation and 90% in the consolidated cohort (p<0.001) (Table 3).

**Table 3:** Compliance with Enhanced Recovery Protocol Interventions Across Study Cohorts. Comparison of patient adherence (%) to specific ERP interventions among historical (pre-ERP), implementation (<70% adherence), and consolidated (>70% adherence) cohorts. Statistical analysis employed generalized Fisher’s exact test for categorical variables. Post hoc pairwise comparisons were performed using two-group Fisher’s exact tests following a significant result in the overall analysis. Superscripts: a: Significant difference (p<0.05) compared to the historical cohort; b: Significant difference (p<0.05) compared to the implementation cohort.

Perioperative interventions	Historical cohort	Implementation cohort	Consolidated cohort	p-value
Preoperative counselling	0%	40%^a^	100%^a,b^	<0.001
Nutritional screening	0%	40%^a^	80%^a,b^	<0.001
Fasting for 2 hours for clear liquids	0%	10%	100%^a,b^	<0.001
Carbohydrate loading	0%	10%	100%^a,b^	<0.001
No bowel preparation	100%	100%	100%	1.000
Antithrombotic prophylaxis	100%	100%	100%	1.000
Preoperative physiotherapy	50%	80%^a^	85%^a^	0.039
Laparoscopic approach	0%	100%^a^	100%^a^	<0.001
Prophylactic antibiotics	100%	100%	100%	1.000
General anesthesia without Neuraxial technique	0%	90%^a^	85%^a^	<0.001
Avoid pelvic drains	0%	60%^a^	85%^a^	<0.001
Prophylaxis for nausea and vomiting	80%	100%	100%	0.029
Early feeding (< 8 hrs.)	50%	55%	90%^a,b^	0.016
Avoid nasogastric tube	100%	100%	100%	1.000
Early mobilization (< 8 hrs.)	0%	35%^a^	90%^a,b^	<0.001
Mean compliance	37%	68%^a^	94%^a,b^	<0.001

**Figure 1:**
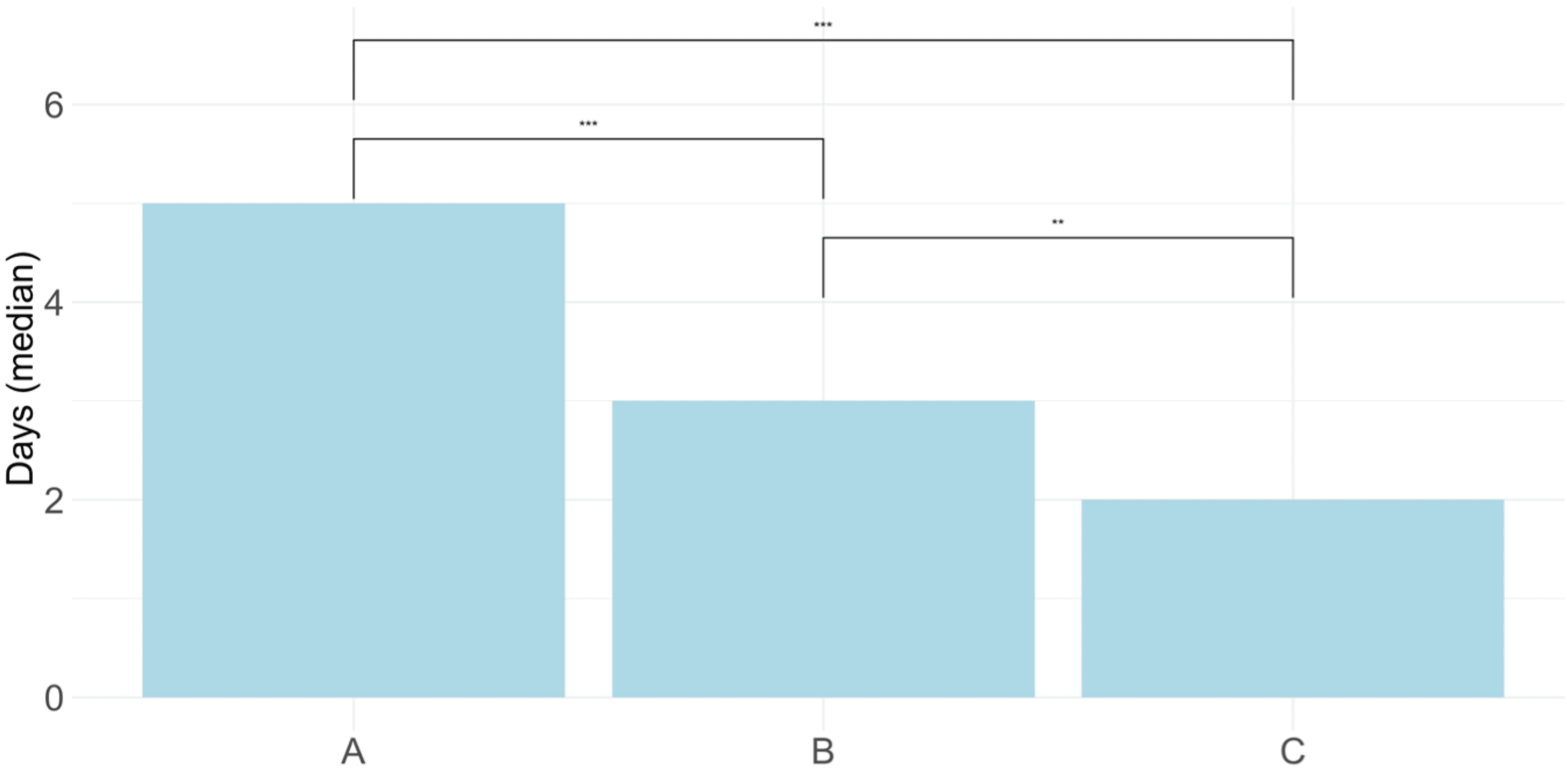
Comparative Analysis of Hospital Length of Stay Across Study Cohorts. Median postoperative hospital length of stay was compared among historical, implementation, and consolidated cohorts. Statistical significance was assessed using Kruskal–Wallis test with subsequent Dunn’s post-hoc analysis. Symbols indicate significant differences: ***p<0.0001; **p<0.001.

Median hospital LOS significantly decreased from five days in the historical cohort to three days in the implementation cohort (p<0.001) and further declined to two days in the consolidated cohort (p<0.001). Notably, a significant reduction was also observed between implementation and consolidated cohorts (p<0.001) (Figure 1).

Complications were systematically evaluated using the Clavien-Dindo classification system. Grade I complications increased numerically from 15% (n=3) in the historical cohort to 30% (n=6) in the implementation cohort and 40% (n=8) in the consolidated cohort, predominantly due to postoperative nausea. This increment, however, did not achieve statistical significance (p=0.213). Grade II complications were exclusively documented in the historical cohort (10%, n=2), while no such complications were observed in the ERP cohorts. Grade III complications occurred significantly less frequently in ERP cohorts compared to the historical cohort (historical 20% vs. consolidated 5%; p=0.008), with no Grade III complications documented in the implementation cohort. Importantly, severe complications classified as Grade IV or V were absent in all cohorts. Additionally, hospital readmissions and reoperations were exclusively observed in the historical cohort (20% each, n=4) (p=0.008) (Table 4).

**Table 4:** Postoperative Complications According to Clavien–Dindo Classification Across Cohorts. Distribution and frequency of postoperative complications classified by Clavien–Dindo grades among the historical, implementation, and consolidated cohorts. Data are reported as numbers (percentage, %). Statistical analysis employed generalized Fisher’s exact test, with subsequent post hoc pairwise comparisons using two-group Fisher’s exact test following significant results in the overall analysis. Superscripts: a: Significant difference (p<0.05) compared to the historical cohort; b: Significant difference (p<0.05) compared to the implementation cohort.

Clavien-Dindo Grade	Complications	Historical cohort	Implementation cohort	Consolidation cohort	p-value
Grade 1	Nausea nº (%)	3 (15)	5 (25)	5 (25)	0.432
	Vomiting nº (%)	0	1 (5)	3 (15)^a^	0.017
Grade 2	Deep Vein Thrombosis nº (%)	2 (10)	0^a,b^	0^a,b^	0.014
	Pneumonia nº (%)	0	0	0	-
	Urinary Tract Infection nº (%)	0	0	0	-
Grade 3	Readmission nº (%)	4 (20)	0^a^	0^a^	0.008
	Reoperation nº (%)	4 (20)	0^a^	0^a^	0.008

## Discussion

This study provides evidence supporting the effectiveness and safety of implementing an ERP tailored specifically for radical prostatectomy. Our analysis demonstrated a significant reduction in hospital LOS associated with the ERP, decreasing from a median of five days in the historical cohort to three days in the implementation cohort and two days in the consolidated cohort. The magnitude of LOS reduction observed aligns closely with findings from previous studies evaluating ERAS protocols in other major abdominal and urological surgeries [5,7,13].

A notable strength of our analysis is the clear demonstration of a “dose-response” relationship between ERP protocol adherence and improved patient outcomes. Patients in the consolidated cohort who achieved a high compliance rate of 94% experienced the greatest LOS reduction, reinforcing the critical importance of protocol adherence. Conversely, the implementation cohort, with a moderate compliance of 68%, showed a smaller, yet significant, reduction in LOS. These results align with findings from Ahmed et al. and Gustafsson et al., which underscored that improved compliance directly enhances postoperative recovery outcomes [14,15].

Interestingly, despite observing longer operative durations in the ERP cohorts, likely associated with the laparoscopic approach, intraoperative blood loss exhibited a substantial decrease compared to the historical cohort. This finding is consistent with existing literature suggesting that laparoscopic techniques inherently contribute to reduced blood loss, lower postoperative morbidity, and enhanced recovery profiles when compared with traditional open surgery [16,17]. Additionally, the ERP cohorts received anesthesia strategies aligned with contemporary ERAS principles, including the avoidance of epidural analgesia in favor of multimodal analgesia. These anesthetic modifications may have contributed positively to faster mobilization and recovery without increasing severe complications, underscoring the importance of anesthesia management in ERP protocols.

One unexpected observation was the higher incidence of mild postoperative complications (Clavien-Dindo Grade I), predominantly nausea, in patients within the ERP cohorts. While statistically insignificant, this increase warrants clinical consideration. Potential explanations include prolonged surgical and anesthesia durations inherent to laparoscopic procedures, increased use of volatile anesthetic agents, and the selective administration of intrathecal morphine, all factors known to potentially exacerbate postoperative nausea and vomiting [5,13,18]. Additionally, adherence to early postoperative feeding guidelines, although beneficial overall, may also contribute to increased gastrointestinal discomfort and nausea. These observations highlight a specific area for protocol refinement, suggesting that more personalized antiemetic strategies and further adjustments to analgesic regimens might enhance patient comfort and satisfaction postoperatively.

Importantly, our findings confirmed the absence of severe postoperative complications (Grades IV-V) in all patient cohorts, with a notable reduction in moderate complications (Grade III), reoperations, and readmissions within ERP cohorts. This outcome aligns with established evidence indicating that ERAS protocols enhance recovery without increasing complication rates [13,16,17]. Our results also resonate with recent meta-analyses demonstrating ERP protocols’ ability to safely reduce LOS and improve postoperative outcomes in prostatectomy patients, reinforcing the importance of protocol adherence and multidisciplinary involvement [5,13].

Despite the strengths highlighted, several limitations should be recognized. Firstly, the retrospective, single-center design limits the generalizability of our results, as institutional practices and team experience heavily influence ERP effectiveness. Prospective, multicenter studies involving larger and more diverse patient populations are needed to validate our findings. Secondly, our center currently lacks robotic-assisted surgical technology, restricting our analysis exclusively to open versus laparoscopic approaches. Although existing literature suggests robotic-assisted radical prostatectomy does not consistently provide superior clinical outcomes compared to laparoscopic approaches [19-22], the global adoption of robotic technology underscores the need for future comparative studies incorporating robotic-assisted procedures within standardized ERP frameworks.

Finally, despite the global trend towards adopting robotic-assisted surgical techniques, economic and infrastructural constraints significantly limit their widespread implementation in low- and middle-income countries. In such resource-limited settings, laparoscopic and open surgical modalities remain predominant due to their economic feasibility and availability of clinical expertise. In this context, ERP implementation- irrespective of the surgical modality employed- represents a practical and cost-effective strategy. Our findings illustrate that ERP effectively optimizes patient outcomes and resource utilization, thus bridging existing disparities in perioperative care until robotic technology becomes more accessible [23,24]. This underscores the universal applicability of ERP protocols and supports their global integration to enhance postoperative recovery and healthcare efficiency, especially in settings where high-cost technological solutions remain out of reach.

## Conclusions

The implementation of a structured, multidisciplinary ERP tailored for radical prostatectomy effectively and safely reduced hospital LOS, decreasing median hospitalization duration from five days in the historical cohort to two days in the consolidated cohort. This significant reduction was achieved without increasing postoperative complications, readmissions, or reoperations. Importantly, our findings underscore a robust correlation between higher ERP compliance and improved patient outcomes, reinforcing the importance of rigorous adherence to enhanced recovery protocols.

The results of this study provide compelling evidence supporting the adoption of ERPs in radical prostatectomy, particularly highlighting their applicability as a cost-effective, globally relevant strategy for enhancing perioperative care. Given the economic and infrastructural constraints that often limit the implementation of advanced robotic surgical technologies, particularly in low- and middle-income settings, ERP represents a pragmatic and broadly accessible approach to optimize perioperative outcomes. Future studies, preferably multicenter and prospective, should further explore ERP implementation across various surgical modalities, including robotic-assisted techniques, to enhance the generalizability and global applicability of these findings.
